# Sleep and Metabolism: An Overview

**DOI:** 10.1155/2010/270832

**Published:** 2010-08-02

**Authors:** Sunil Sharma, Mani Kavuru

**Affiliations:** Division of Pulmonary, Critical Care and Sleep Medicine, Department of Internal Medicine, Brody School of Medicine, Greenville, 27834 NC, USA

## Abstract

Sleep and its disorders are increasingly becoming important in our sleep deprived society. Sleep is intricately connected to various hormonal and metabolic processes in the body and is important in maintaining metabolic homeostasis. Research shows that sleep deprivation and sleep disorders may have profound metabolic and cardiovascular implications. Sleep deprivation, sleep disordered breathing, and circadian misalignment are believed to cause metabolic dysregulation through myriad pathways involving sympathetic overstimulation, hormonal imbalance, and subclinical inflammation. This paper reviews sleep and metabolism, and how sleep deprivation and sleep disorders may be altering human metabolism.

## 1. Introduction

Consequences of sleep deprivation and fragmentation are being increasingly recognized. We are a sleep deprived society with evidence showing that we sleep on an average 6.8 hours as opposed to 9 hours a century ago. Around 30% of adults report sleeping less than 6 hours per night [1–3]. The 24/7 economy and its subsequent impact on sleep patterns may be testing the bodies limits to maintain metabolic and hormonal equilibrium. Prevalence of both diabetes and obesity has increased to acquire pandemic proportions. Though other factors such as diet and reduced physical activity have contributed to the obesity epidemic the impact of sleep dysregulation on causing metabolic derangements is being increasingly recognized. Considering only a small percentage of people can maintain a healthy weight over a long period on diet and exercise alone, the impact of sleep on weight has opened a new venue for potential intervention.

Understanding this topic is important as both sleep and metabolic dysregulation are common and growing problems. There are many unresolved issues including cause and effect, pathogenesis and potential implications to therapy.

## 2. Metabolism in Normal Sleep

Human sleep comprises of nonrapid eye movement sleep (NREM) and REM sleep. NREM is further comprised of three stages (stages N1, N2, and N3). N3, also referred to as slow wave sleep, is considered deep sleep with the body being least metabolically active during this period. REM sleep is characterized by vivid dreams, loss of muscle tone, and rapid eye movements. The EEG pattern of REM sleep closely mimics that of wakefulness marked by a high-frequency and low-voltage wave pattern. NREM and REM sleep occur alternatively in cycles of around 90 minutes throughout the night [4]. The first half of the night is predominantly NREM, and the second half is predominantly REM sleep. Sleep architecture, though, is heavily influenced by genetic and environmental factors including sex, race, socioeconomic status and culture among others. Sleep duration in mammals generally depends on the size of the animal [5]. Elephants require only 3 hours of sleep while rats and cats can spend up to 18 hours in sleep. It is postulated that this may be due to differences in metabolism. Smaller animals have higher metabolic rate and higher body and brain temperatures compared to larger animals.

 Metabolism is defined as the whole range of biochemical processes that occur within a living organism. It constitutes the two processes of anabolism (build up) and catabolism (break down). In simpler terms, metabolism is the amount of energy (calories) the body burns to maintain itself. Metabolism in general is associated with cell injury due to the release of free radicals [6]. The lower metabolic rate and brain temperature occurring during non-REM sleep seem to provide an opportunity to deal with the damage done during awake and metabolically active period. Siegel and his group from University of California at Los Angeles (UCLA) have shown brain damage in sleep-deprived rats [7]. Most data available and referred to in this review deals with glucose utilization and energy expenditure.

It is believed that during normal sleep the metabolic rate reduces by around 15% and reaches a minimum in the morning in a standard circadian pattern [8, 9]. Only a 15% reduction in metabolic rate appears counter-intuitive considering the prolonged state of physical inactivity. However, the basal metabolic rate constitutes 80% of the metabolism needed to maintain all cellular processes in the body. Glucose utilization in normal subjects is highest during wakeful state and lowest in NREM sleep and intermediate in REM sleep [10].

Growth hormone and cortisol are two hormones that have an impact on glucose regulation. Growth hormone is typically elevated at onset of sleep with highest levels during slow wave sleep (SWS) while cortisol levels are greatly increased during the second half of the sleep, predominantly in REM sleep [11, 12]. Studies on normal subjects with constant glucose infusion during sleep (to suppress endogenous glucose production) have revealed that a fall in brain glucose metabolism contributed to a two-thirds fall in systemic glucose utilization during sleep despite increase in glucose and insulin levels. Reduced muscle tone and anti-insulin like effect of growth hormone surge during the first half of sleep contributes to the rest of fall in glucose utilization [13]. Hence there is a relative state of insulin resistance during early phases of sleep.

During the latter part of sleep the glucose and insulin levels fall despite continuous infusion of glucose. Other studies have shown similar findings suggesting increased glucose utilization during REM phase of the sleep and increased glucose levels in the evening with reduced insulin sensitivity [13]. In addition, studies have shown an increase in cortisol levels in evening after just one night of sleep deprivation contributes to glucose dysregulation [14].

## 3. Consequences of Sleep Deprivation

Although impact of sleep on glucose regulation has been known and studied for some time, metabolic dysregulation with sleep loss has only recently been understood. Prior research models had focused on acute sleep deprivation. Studies done by Hampton et al. revealed that when subjects were made to simulate shift work it resulted in alterations in postprandial glucose and lipid metabolism [15]. This response was noted with 9-hour phase advance. The same group later showed that it takes at least 2 days to adapt to eating meals on a simulated night shift [16]. Since the body has good rebound capacity, the metabolic derangements, if any, were readily corrected in acute sleep loss model. The more practical model to study is recurrent prolonged partial sleep deprivation, which mirrors real life scenarios. In fact, studies have shown that both slow wave sleep (SWS) and growth hormone (GH) rebound after acute sleep loss, but no such spike is seen in SWS and GH during recurrent partial sleep restriction [17, 18]. For these reasons, chronic sleep deprivation models are more relevant in terms of clinical significance and subject of our focus.

Though a previous study has shown reduced insulin sensitivity to oral glucose administration it was limited to one night of sleep deprivation [19]. The first detailed study to examine the impact of partial sleep deprivation on glucose tolerance was performed by Cauter et al. at the University of Chicago. Eleven healthy young men were subjected to 4 hours in bed for 6 nights followed by 12 hours for 7 nights to recover from sleep debt. Intravenous glucose tolerance test was performed on the sixth day. Sleep deprivation resulted in reduced glucose tolerance (rate of glucose clearance) by 40%. Glucose effectiveness, a measure of noninsulin dependent glucose disposal, was 30% reduced along with a reduction in insulin response to glucose [20]. A subsequent study with a randomized crossover design by the same group confirmed the findings [21]. The conclusion from these laboratory based studies is that a week of sleep deprivation can result in a significant alteration in metabolic and endocrine function. 

The mechanism of sleep deprivation causing metabolic dysregulation may be multifactorial. Changes in hormonal secretion profile as discussed above may have profound effect on glucose regulation [13]. 

Sympathetic stimulation has been shown to occur with sleep deprivation [22] and might contribute in the metabolic dysregulation. The third possible mechanism is inflammation. Experimental sleep deprivation has been found to alter immune response and increase proinflammatory markers such as IL-6, TNF-  *α*, and CRP [23–25]. 

## 4. Sleep Duration and Risk of Diabetes

It is projected that by 2010, 221 million people would be affected by diabetes globally [26]. It is important we understand the role of sleep in glucose metabolism and potential directions for new research and therapy.

Epidemiological data increasingly suggests that short sleep duration or chronic partial sleep deprivation may increase the risk of type II diabetes. In a large cohort of nurses (Nurse Health Study with more than 70,000 respondents), self-reported short (5 hours or less) and long duration of sleep (9 hours or more) was associated with symptomatic diabetes with a relative risk of 1.34 for short [1.04–1.72]) and long 1.35 for long [1.04–1.75]) sleepers [27]. A Swedish study with more than 2000 people followed for over 10 years revealed that short duration of sleep (< 5 hours) and difficulty initiating and maintaining sleep were associated with higher incidence of diabetes in men (but not in women) even after adjusting for confounding factors like age, BMI, snoring, depression, and hypertension [28]. In another study by Yaggi and colleagues, a large cohort of men from the Massachusetts Male Aging Study (MMAS), without diabetes at baseline was followed for more than 15 years in a longitudinal study. Subjects who self-reported less than 6 hours of sleep were twice as likely to develop diabetes. Subjects sleeping longer than 8 hours were three times more likely to develop diabetes. This elevated risk remained after adjusting for HTN, age, waist circumference, smoking, and education [29].

Although epidemiological studies do not establish causality, these studies are consistent with the physiological data discussed earlier. 

In summary, the laboratory data seem to be supported by large epidemiological studies (including longitudinal) that short sleep duration might play an important role in altering glucose metabolism. However, these results appear to be more applicable to men than women for reasons not fully understood. The relationship between increased sleep duration and risk for diabetes is not fully understood.

## 5. Sleep Loss and Appetite

### 5.1. Leptin and Ghrelin

The appetite center is believed to be located in the arcuate nucleus of the hypothalamus, which in turn is influenced and regulated by peripheral hormones such as leptin and ghrelin. Leptin is an appetite suppressant hormone produced by adipose tissue, and ghrelin is released from the stomach primarily in response to fasting and promotes the feeling of hunger [30]. Leptin has been shown to rapidly increase or decrease in response to caloric shortage or surplus [31]. In human studies, a marked rise in leptin and ghrelin are noted during sleep, though the levels of ghrelin tend to fall during latter part of night despite maintenance of fasting conditions [32, 33]. It is believed that leptin levels stay elevated due to melatonin-influenced insulin-triggered leptin production [34]. This suggests the effects of the rising ghrelin levels during the early part of night might be blunted by leptin, preventing arousal during sleep due to hunger. Spiegel and colleagues work on healthy humans have also shown that sleep deprivation lowered leptin levels by 19% compared to sleep extension [35]. They further observed that sleep deprivation blunted the diurnal variation normally seen without sleep deprivation. These findings were confirmed by the same group in a randomized crossover trial of sleep restriction in normal human subjects. Subjects were sleep restricted for 2 nights (4 hours/night) followed by 2 nights of sleep compensation (10 hours/night) while receiving continuous glucose infusion. Significant reduction in leptin levels (18%) were noted with a concomitant 28% increase in ghrelin levels [36]. A 24% increase in hunger rating and 23% increase in appetite rating were also noted. Reduction of leptin level was a significant predictor of magnitude of hunger observed. Further analysis of appetite rating revealed that subjects tended to show more preference to high carbohydrate foods (sweets, salty food and starchy foods), that is, Craving for salty food increased by 45% (*P* = .02). This suggests that sleep deprivation may affect eating behavior favoring nonhomeostatic food intake (food intake driven by emotional/psychological need rather than caloric need of the body) [36]. Acute sleep deprivation of single night in young healthy men increases ghrelin levels but not leptin levels [37]. Sleep deprivation may also affect the circadian profile of leptin. Healthy men subjected to 88 hours of sustained wakefulness have been shown to have reduced diurnal amplitude of leptin, with return to normal rhythm on sleep recovery [38]. Recent study by Penev et al. revealed that short, partial sleep deprivation (< 5.5 hours/day) in normal subjects resulted in increased consumption of calories from snacks but no increase in total energy consumption. This study did not show any significant changes in leptin or ghrelin levels. The authors hypothesized that the higher carbohydrate intake due to sleep restriction may be due to prolonged exposure to more palatable food [39].

A population based study of 1024 patients (derived from the Wisconsin sleep cohort study, a large longitudinal population based study on sleep disorders) that revealed similar alteration in leptin and ghrelin levels based on total sleep time as measured by overnight polysomnography [40]. The study also revealed that chronic sleep deprivation (sleep less than 8 hours) was associated with increase in BMI.

Leptin has been found to be elevated in obese individuals and patients with obstructive sleep apnea. It is believed that the elevated CRP levels in obesity and obstructive sleep apnea bind to leptin resulting in elevated serum levels [41]. This is believed to be accompanied by leptin resistance due to down-regulation of leptin receptors. This leads to impairment in weight regulation and may contribute to weight gain [42]. 

The fact that relationship between sleep and leptin may be bidirectional is evident by animal studies by Laposky et al. Leptin deficient mice have been shown to have more disrupted sleep architecture, increased time spent in NREM sleep and increased total sleep time [43]. The same authors also showed that leptin signaling exerts a role in sleep-wake regulation. Obese/diabetic mouse with mutation of leptin receptors exhibited sleep fragmentation, decreased compensatory response to sleep deprivation and decrease locomotor response [44]. 

In summary, leptin may represent an important link between, sleep, circadian rhythm and metabolism.

### 5.2. Orexins

Discovery of excitatory neuropeptide hormones orexins A and B (hypocretins) expressed from neurons located in perifornical region of the hypothalamus, [45] has significantly added to our knowledge. Energy homeostasis, as determined by the balance between calorie intake and energy expenditure, is regulated by hypothalamus [46]. Orexins neurons are located in the hypothalamus and from them project throughout the brain, including paraventricular nucleus of the thalamus, the arcuate nucleus and, most notably, the locus coeruleus, dorsal, and tuberomammillary nucleus (areas involved in wakefulness) but not the cerebellum [47, 48]. Orexins have been found to be influenced by peripheral metabolic cues like leptin, ghrelin, and glucose which indicated that orexins may provide an important link between sleep and metabolism [49] and play a key role in metabolism. Administration of orexins increases food intake and stimulates wakefulness and energy expenditure [50, 51]. Narcolepsy, a sleep disorder caused by orexin deficiency is accompanied by decreased energy intake, increased BMI, and increased incidence of type 2 diabetes [52, 53]. Orexin knockout mice also demonstrate late onset weight gain [54]. The fact that orexin deficient mice display reduced energy expenditure independent of sleep duration and wake durations suggests that orexin-induced increased metabolism is not simply due to its wake promoting action and subsequent more exposure to food [55]. Recently investigators, working on glucose metabolism in orexin knockout mice, found that orexin is essential for maintenance of normal insulin sensitivity with increasing age [56]. In conclusion, these findings suggest that sleep deprivation may blunt and upset the finely tuned signaling response of hormones to body's caloric needs not only leading to increase in appetite but also a propensity for psychological eating (nonhomeostatic food intake).

## 6. Sleep Deprivation and Weight

More than two dozen epidemiological studies from around the globe looking at sleep deprivation and BMI in humans have shown association between decreased obesity and an increase in sleep duration. These studies however do not establish a causal relationship. Few studies revealed a U-shaped curve with lowest mean BMI associated with 7.7 hours/night [40, 57, 58]. NHANES study revealed (using a normal benchmark of 7 hours/night) odds ratio for obesity as 2.35 for 2–4 hours/night, 1.60 for 5 hours/night, and 1.27 for 6 hours/night of sleep. This association was observed in both obese and nonobese subjects and adjustment for sex, age, and population size though the relationship appeared to wane with age [40, 57, 59–62]. These findings are supported by studies conducted in children [63–65]. The impact of short sleep appeared to be greatest in children and young adults as compared to older adults [61–69]. 

A major limitation of epidemiological studies looking at sleep duration and BMI has been self-reporting of sleep time as opposed to objective measurement. However recent studies [56, 70–72] have looked at objectively measuring sleep via actigraphy (device worn like a watch with ability to record gross motor movements) and overnight polysomnography. The CARDIA study [70] used a large cohort of subjects and obtained 3 nights of actigraphy. Mean sleep duration was found to be 6.1 hours with variation among different race-sex groups (mean sleep duration of 6.7 hours in white females to 5.1 hours in African-American males). This study also found moderate correlation between subjective and objective sleep time duration though participants overreported their sleep duration by about 0.8 hours (measured sleep duration was 6 hours versus a self-reported time of 6.8 hours). The Danish revealed that sleeping less than 5 hours was associated with higher BMI in elderly population. Sleep fragmentation was also found to be strongly associated with increased BMI in this study [71]. In another recent study with over 3000 patients where the sleep duration was again objectively recorded by actigraphy, researchers found that older men and women with reduced amounts of sleep (less than 5 hours) as measured by actigraphy had an elevated BMI. Sleeping 5 or fewer hours per night was associated with 3.7-fold greater odds of obesity among men and 2.3-fold increase among women compared to those sleeping 7-8 hours per night Patel et al. [73]. Apart from cross sectional studies, there have been 9 prospective/longitudinal studies in adults and children, 8 of which have shown similar findings of sleep deprivation and a higher prevalence of obesity [61, 62, 65, 74–76].

Data regarding impact of sleep deprivation on weight loss is conflicting in animals and humans. Sleep deprivation in rodent models causes weight loss despite hyperphagia [63–68]. These differences in rodents and humans may be explained by increased brown fat in rodents (rarely present in adult humans), which is metabolically more active and has been shown to increase thermogenesis and total energy expenditure [67]. In conclusion, epidemiological data is suggestive of weight gain with sleep deprivation though a few studies have also noted weight gain with prolonged sleep. Based on data on sleep duration and weight, sleep hygiene counseling could form an important tool in management of obesity.

## 7. Obstructive Sleep Apnea and Type II Diabetes

Obstructive sleep apnea (OSA) is a highly prevalent disorder affecting 2%–4% of the population. It is characterized by intermittent but repetitive cessation of breathing accompanied by hypoxemia or reduced level of oxygen in blood. OSA has significant affect on sleep architecture including sleep fragmentation and reduction in stage REM and slow wave sleep (SWS) [77].

Data from a recent national survey shows that as many as one in four adults are at risk of having OSA [78]. More than 50% of patients with type II diabetes have obstructive sleep apnea [79]. Studies as early as 1985 had noted an association between snoring, diabetes, and abnormal glucose tolerance [80, 81]. A Swedish study with longitudinal design and over 2600 subjects revealed habitual snoring as an independent risk factor for diabetes at 10 year followup [82]. Several studies since then have supported the findings of snoring associated with increased prevalence of type II diabetes, with habitual snorers being at twice the risk for having diabetes [83, 84]. 

Cross-sectional studies using polysomnography confirmed OSA have similarly shown increased insulin resistance, glucose intolerance and an increase in HgA1C [85–88]. Importantly, the severity of OSA appears to be proportional to the severity of metabolic dysfunction. This association stood after adjustment to age, sex, and adiposity. However, a longitudinal study by Wisconsin sleep cohort group failed to show an independent relationship between OSA and incidence of diabetes at 4-year followup. These conflicting results may be due to short duration of the study [89].

Clinic-based studies have demonstrated similar trends favoring an association between OSA and diabetes. In a study of patients with OSA compared to obese patients without OSA were found to have higher fasting glucose, higher insulin levels, and higher systemic inflammatory markers [90]. In a subsequent larger study by Punjabi and colleagues, 150 mildly obese but otherwise healthy men underwent polysomnography, oral glucose tolerance test, and determination of body fat. In this study, the prevalence of OSA (defined as AHI > 10/hrour) was more than 45%. After adjusting for BMI, OSA was associated with increased risk of having impaired glucose tolerance (Odds ratio of 2.15) and related to degree of oxygen desaturation [85]. Studies on a large Asian cohort done by Ip et al. also found OSA to be independently associated with insulin resistance as measured by HOMA-IR (homeostasis model assessment of insulin resistance) [86]. Similarly a large European study (595 patients) revealed that type II diabetes was present in 30% of patients with OSA [91] and a Japanese study (213 patients) found increased insulin resistance in patients with OSA [92]. Though most studies looked at BMI, few studies looked at visceral obesity and waist to hip ratio, which more closely relates to insulin resistance than BMI [92, 93]. Though most studies have supported an association between OSA and diabetes/glucose dysregulation, a few studies have been negative [94, 95]. This is not surprising as obesity is a huge confounding factor in all studies dealing with OSA. These data simply indicate an association between OSA and type II diabetes and does not however establish causality or direction of causality. 

If it is true that OSA causes diabetes, then treatment of OSA should mitigate the metabolic dysregulation. However, treatment by CPAP (continuous positive airway pressure therapy), which is currently the most accepted therapeutic intervention for OSA has shown inconsistent results. Several studies have shown improvement in insulin sensitivity after varying periods of CPAP therapy in patients with diabetics and nondiabetics [96–98], including a study showing a reduction in HbA1C [99]. A German study using the hyperinsulinemic euglycemic clamp technique (the gold standard for measuring insulin sensitivity) evaluated 40 patients with moderate to severe obstructive sleep apnea and found improved insulin sensitivity after only 2 days of CPAP therapy, which persisted during a 3 month followup [100]. This rapid improvement suggests that the resolution of sympathetic drive might play an important role in the pathogenesis of metabolic dysregulation seen in patients with obstructive sleep apnea. However, the debate on the impact of CPAP therapy in mitigating metabolic dysregulation is far from resolved. Equal numbers of studies have shown no impact of therapy on diabetes or glucose metabolism [101–104]. A uniform problem with these studies has been small number of patients and no controls. Recently, two randomized controlled trials have been conducted. A study by Coughlin et al. took 34 patients with severe sleep apnea and metabolic syndrome and randomized them to receive CPAP therapy versus sham CPAP followed by a crossover after 6 weeks. The study failed to show any improvement in insulin sensitivity or metabolic profile despite improvement in blood pressure [105]. West et al. evaluated 42 patients with OSA and diabetes and randomized them to 3 months of treatment versus sham treatment. The study did not show any significant improvement in glycosylated hemoglobin or insulin resistance measured by euglycemic clamp and HOMA [106]. The compliance in this study was however suboptimal (3.6 hours/night) and may have affected the outcome. Whether this had any impact is debatable. One major confounding factor in these studies is obesity. Harsh and colleagues showed that the improvement in insulin sensitivity in patients with BMI > 30 kg/m^2^ is minimal [100] but improved after 3 months [107]. Future studies are required to define the correct patient profile, time duration and impact of compliance, to get better understanding of the role of CPAP therapy in improving diabetes.

**Figure 1:**
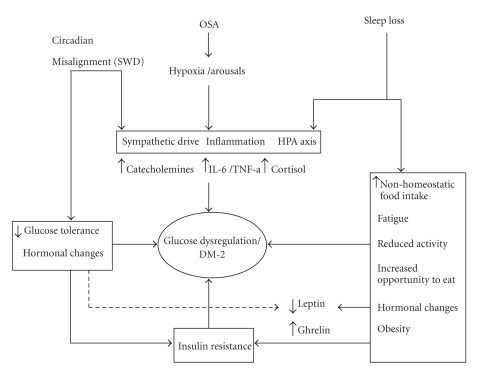
Schematic diagram of potential mechanism of glucose dysregulation/diabetes pathogenesis, secondary to sleep loss, sleep apnea, and circadian misalignment. The three main sleep disorders are listed at the top. Arrows point to possible pathophysiological alteration the disorders may induce. Some of the pathways are common to all the disorders and are listed together, that is, sympathetic drive, inflammation, and alteration of HPA axis. Sleep loss may in addition lead to changes like hormonal imbalance and reduced activity (listed on the box to the right of the diagram). Similarly circadian alteration may also cause insulin resistance and hormonal imbalance (shown in the box to the left). All these pathophysiological alterations eventually may lead to type II diabetes which is shown in the center. SWD: Shift work disorder.

It is believed that obstructive sleep apnea may cause metabolic dysregulation through several pathways. Sympathetic surge is known to occur with each apnea event. Sympathetic activation has been shown to increase levels of circulating free fatty acids because of the stimulation of lipolysis, which promotes insulin resistance [86]. Elevated catecholamine levels were found in people with more wake time after sleep onset [108]. Additionally, sleep fragmentation, recurrent hypoxemia, and triggering of inflammatory cytokines on a nightly basis may all contribute to higher propensity to metabolic syndrome and type II diabetes. The relative contribution of any of the above pathways is not known. Some of these pathways may overlap with suggested pathophysiological pathways of sleep deprivation and circadian misalignment (Figure 1).

In summary while there is growing evidence of an association between OSA and metabolic dysregulation the direction of causality and decoupling of major confounding factor of adiposity has not been clearly stated. Data on interventional studies is also conflicting and marred by small sample sizes, inadequate power, and observational design.

## 8. Metabolic Consequences of Shift Work Disorder

Sleep is controlled by two powerful processes: circadian and homeostatic [109]. During waking hours, the sleep drive gradually increases until it reaches a critical threshold. This drive is referred to as homeostatic. Circadian rhythm, on the other hand, is a signal generated by the master clock, the suprachiasmatic nucleus (SCN) located in the anterior hypothalamus. Circadian rhythm, derived from the Latin term *“circa diem” *which literally means “approximately one day” is the body's internal clock. This clock is set at slightly over 24 hours. It controls sleep as well as most biological processes, including hormone production, metabolism, core body temperature variations, and cell regeneration among others [110]. This clock is normally highly synchronized to environmental cues (Zeitgebers, German for “time giver”), the strongest of them being the light-dark cycle. In most humans the alertness pattern shows a biphasic distribution, with a mid-day decrease in alertness around 2–4 pm, followed by an increased alertness during mid to late evening, and finally declining to its lowest levels during the night [109, 111]. Almost all physiological systems in humans run slightly over a 24 hour cycle. Disturbance of this well-regulated circadian rhythm and homeostatic drive (circadian misalignment) can lead to various sleep disorders collectively known as circadian rhythm sleep disorders. Shift work disorder is one of the circadian rhythm disorders which have been a focus of large epidemiological studies for its potential implications on health.

People involved in some form of shift work are increasing globally. U.S. Bureau of Labor statistics indicate that 8.6 million people were shift workers in 2004 in the United States alone [112]. Circadian misalignment due to shift work or jet lag has been associated with obesity, diabetes, and cardio-vascular diseases. A Swedish study followed shift workers for 15 years and reported increased relative risk for ischemic heart disease (RR = 2.8) as compared to daytime workers independent of smoking and age with similar socioeconomic background [113]. More recent studies have suggested that shift work is the most significant source of ischemic heart disease accounting for over 10% of mortality in men and over 5% in women [114]. Another prospective study found increased risk of circulatory diseases in shift workers after controlling for confounding factors [115]. In a large cohort of subjects followed prospectively in the Nurse Health Study II investigators found increased risk of type II diabetes in young and middle age nurses working in rotating night shift work [116]. Cross-sectional data also suggests higher triglyceride levels, lower HDL levels, and more obesity in the shift workers than daytime workers [117, 118]. Another recent study emulating shift work for short duration (10 days) revealed decreased leptin, increased glucose, and reversed cortisol rhythm. Circadian misalignment caused 3 of the 8 subjects to exhibit postprandial glucose responses in the range typical of a prediabetic state [119].

Mechanistic pathways by which shift work can cause metabolic dysregulation are not clear but appear to involve hormonal alterations and increased sympathetic drive leading to decreased insulin sensitivity and insufficient beta-cell compensation. Possibility of altered melatonin profile during circadian misalignment contributing is another potential pathway as there is some evidence that melatonin may inhibit glucose-induced insulin release [120]. Additionally, animal models subjected to circadian alterations simulating shift-work have resulted in premature death [121]. 

In conclusion, the limited data regarding circadian misalignment suggests its role, albeit unclear, in metabolic dysregulation. Since sleep deprivation is commonly associated with shift work disorder further prospective trials adjusting for sleep deprivation are required to establish role of circadian misalignment as opposed to indirect effect of sleep deprivation on metabolic dysregulation.

## 9. Energy Expenditure in Sleep and Sleep Disorders

Several studies have looked at the relationship of body energy expenditure in sleep and sleep disorders (mainly OSA). The energy expenditure of human body appears to reduce and is lowest during sleep [122–125]. This reduction in energy expenditure may be influenced by circadian rhythm [122, 126], changes in body temperature [127], and reduction in muscle activity [128, 129], not to mention the depth and duration of sleep and physical activity [8, 129–132]. Energy expenditure has also been reported to vary depending on the stage of sleep [8, 133]. Race also seems to play a role as African-Americans appear to have a lower sleep metabolic rate (SMR) and increased propensity for weight gain as compared to Caucasians [134]. Also SMR decreases during sleep as a function of BMI and the decrease rate in SMR is larger as BMI increases [134].

Acute sleep loss results in small increase in SMR [122, 133, 134]. A similarly small increase in SMR has been recorded in chronic sleep deprivation [135].

Limited studies looking at energy expenditure in patients with obstructive sleep apnea have revealed mixed results. Stenlof et al. found higher total energy expenditure (TEE) and SMR with reduction in energy expenditure upon treatment with continuous positive pressure therapy (CPAP). A study by Lin et al. found increase in SMR but not morning BMR in patients with OSA. Patients in this study who underwent laser-assisted uvulopalatoplasty demonstrated a reduction in SMR. However, Hins et al. found no relationship between OSA and TEE or SMR. Similar mixed results have been noted in children [136–138].

In summary, energy expenditure is reduced during sleep. Sleep deprivation appears to increase energy expenditure. Data in impact of sleep apnea on energy expenditure is equivocal. Studies in patients with OSA are also limited by small size and lack of body composition data which significantly impacts energy expenditure. It is also important to point out that most of these studies have utilized indirect calorimetry technique instead of the gold standard metabolic chamber (direct calorimetry). Larger studies utilizing direct calorimetric techniques are needed to understand the impact of sleep apnea on energy expenditure. 

## 10. Conclusions

Sleep disorders and diabetes are rapidly growing problems with grave public health implications. There is growing interest and evidence that sleep loss and sleep disorders have a significant impact on metabolism. Laboratory studies have clearly shown that sleep deprivation can alter the glucose metabolism and hormones involved in regulating metabolism, that is, decreased leptin levels and increased ghrelin levels. A majority of large epidemiological studies have suggested that chronic partial sleep deprivation is associated with an increased risk of obesity and diabetes. However, there are several areas where the data conflicts. The role of gender is not entirely clear. Ayas et al. and Mallon et al. have shown that while sleep duration does predict diabetes in women, the significance is lost once corrected for risk factors like BMI. The relationship of sleep duration to metabolic dysregulation is also found to be U-shaped in many studies (nurse health study, sleep heart health study, and Massachusetts male health study) suggesting that not only short duration but also longer duration may have the potential to disturb the metabolic equilibrium of the body. Paradoxically a similar U-shaped relation is also noted in several studies looking at the relationship between sleep and weight, with both short and long sleep leading to weight gain [62, 139]. Most epidemiological studies have relied on subjective self-reported measures of sleep duration. 

Further studies are needed to clearly elucidate the role of gender, sleep duration, and metabolism with more objective measurements of sleep. Also needing to be clarified is the difference between sleep deprivations due to voluntary sleep loss versus Insomnia. A model of nonobese patients with OSA may help decouple the impact of adiposity on diabetes. Differences exist between human and animal response to sleep deprivation on weight. Mechanism explaining the complex interaction between sleep and metabolism need to be further explored if we hope to derive more clinical mileage with sleep becoming an important tool to fight the obesity pandemic.
